# A Pathophysiology-Integrated Nomogram to Predict Tricuspid Regurgitation Progression After Isolated Mitral Valve Surgery: A Retrospective Cohort Study

**DOI:** 10.31083/RCM46780

**Published:** 2026-05-06

**Authors:** Yichao Pan, Yuxin Kang, Huan Lin, Zhijian Lin, Ling Chen

**Affiliations:** ^1^Department of Anesthesiology, Zhangzhou Affiliated Hospital of Fujian Medical University, 363000 Zhangzhou, Fujian, China; ^2^Department of Cardiac Surgery, Zhangzhou Affiliated Hospital of Fujian Medical University, 363000 Zhangzhou, Fujian, China; ^3^Department of Cardiac Surgery, Fujian Medical University Union Hospital, 350001 Fuzhou, Fujian, China

**Keywords:** functional tricuspid regurgitation, mitral valve surgery, disease progression, prediction model, nomogram, decision curve analysis, risk stratification

## Abstract

**Background:**

Functional tricuspid regurgitation (FTR) frequently progresses after isolated mitral valve (MV) surgery in patients with pre-existing annular dilation, leading to adverse long-term outcomes. Notably, current guideline recommendations for concomitant tricuspid valve intervention, based primarily on annular size, lack precision for individualized risk prediction and may lead to both overtreatment and undertreatment.

**Methods:**

This retrospective, single-center cohort study included 398 patients with mild FTR and tricuspid annular dilation (>40 mm) who underwent isolated MV surgery (2010–2018). The primary endpoint was progression to at least moderate tricuspid regurgitation (TR) on the follow-up echocardiography. Multivariable logistic regression identified independent predictors. A nomogram was developed and internally validated via bootstrapping. Model performance was assessed using discrimination (area under the curve [AUC]), calibration (calibration plots and mean absolute error), and clinical utility (decision curve analysis [DCA] and clinical impact curve [CIC]).

**Results:**

TR progression occurred in 119 patients (29.9%) over a median follow-up of 5.2 years (interquartile range: 3.1–7.4). Multivariable analysis identified four independent preoperative predictors: atrial fibrillation type (paroxysmal: odds ratio [OR] 2.764, 95% confidence interval [CI] 1.682–4.532; persistent: OR: 3.422, 95% CI: 2.081–5.625; permanent: OR: 2.345, 95% CI: 1.404–3.917; all *p* < 0.001), tricuspid annular diameter index (per 1 mm/m^2^ increase: OR: 2.531, 95% CI: 1.767–3.649; *p *< 0.001), pulmonary artery systolic pressure (per 5 mmHg increase: OR: 3.246, 95% CI: 2.191–4.800; *p *< 0.001), and left atrial volume index (per 5 mL/m^2^ increase: OR: 1.876, 95% CI: 1.287–2.733; *p *= 0.001). The resulting nomogram demonstrated good discrimination, with an optimism-corrected AUC of 0.732 (95% CI: 0.701–0.763) following internal validation with 1000 bootstrap resamples (apparent AUC: 0.744; 95% CI: 0.712–0.797). The model also showed excellent calibration (calibration slope: 0.94; calibration-in-the-large: –0.03; Brier score: 0.152). DCA confirmed a superior net benefit relative to treat-all or no treatment strategies across threshold probabilities of 20–70%, with an optimal threshold of 45% for clinical decision-making.

**Conclusions:**

This study developed and internally validated a pathophysiology-integrated nomogram accurately predicting the risk of TR progression after isolated MV surgery. This tool, which incorporates readily available preoperative variables, facilitates personalized risk stratification and evidence-based decision-making regarding concomitant tricuspid intervention, thereby potentially optimizing long-term outcomes for patients with functional tricuspid regurgitation.

## 1. Introduction

Functional tricuspid regurgitation (FTR) is a prevalent and consequential sequela of left-sided valvular heart disease, most commonly mitral valve pathologies [1]. Its pathogenesis is multifactorial, primarily driven by tricuspid annular (TA) dilation, right ventricular remodeling, and leaflet tethering forces secondary to chronic pulmonary hypertension and right ventricular volume overload [2, 3]. While successful mitral valve (MV) surgery ameliorates left atrial pressure and reduces pulmonary artery systolic pressure (PASP), potentially mitigating functional tricuspid regurgitation (TR), the intrinsically dilated and remodeled tricuspid apparatus often fails to reverse adequately. This persistent maladaptive remodeling can lead to the progression of initially mild FTR, resulting in significant long-term morbidity, including right heart failure, increased rehospitalization rates, and mortality [4, 5, 6].

The 2021 ESC/EACTS guidelines recommend concomitant tricuspid surgery for severe FTR (Class I) and suggest it for mild-to-moderate FTR with annular dilation (≥40 mm or >21 mm/m^2^; Class IIa, Level B) [7]. While representing a significant update from prior consensus-based recommendations [8, 9], this anatomical criterion remains contentious. Proponents for a more aggressive approach argue that prophylactic tricuspid surgery prevents inevitable TR progression, avoids the high risk of reoperation, and improves long-term outcomes [10, 11]. Conversely, opponents caution against universal concomitant tricuspid valve annuloplasty, arguing that subjecting all patients with annular dilation to repair unnecessarily exposes those whose TR would not progress to the procedure’s inherent risks, a concern supported by long-term data on repair failure [12, 13].

This critical knowledge gap underscores the pressing need for a more nuanced, individualized risk stratification tool. Identifying which patients with mild FTR and annular dilation are at the highest risk for progression post-MV surgery is paramount for optimizing surgical strategy—reserving concomitant tricuspid surgery for those most likely to benefit while sparing others its associated risks.

To address this unmet clinical need, we conducted a large-scale, retrospective cohort study of patients with mild FTR and tricuspid annular dilation (tricuspid annular diameter [TAD] >40 mm) undergoing isolated MV surgery. We hypothesized that specific preoperative echocardiographic and clinical markers could reliably predict the risk of postoperative TR progression. Utilizing statistical modeling and internal validation techniques, we aimed to develop and validate a clinically applicable predictive nomogram. This tool is designed to facilitate personalized surgical decision-making, ultimately aiming to improve long-term clinical outcomes by identifying high-risk patients who might benefit from a concomitant tricuspid procedure during the initial mitral surgery.

## 2. Materials and Methods

### 2.1 Study Design and Population

This single-center, retrospective cohort study was conducted to investigate the progression of mild FTR and identify its predictors in patients with dilated tricuspid annulus undergoing isolated MV surgery. The study adhered to the principles of the Declaration of Helsinki and was approved by the Institutional Review Board (IRB) of Zhangzhou Affiliated Hospital of Fujian Medical University. The requirement for individual informed consent for this retrospective analysis was waived by the Ethics Committee due to the retrospective nature of the study and the use of anonymized data.

We consecutively screened all adult patients (≥18 years) who underwent isolated mitral valve surgery (repair or replacement) for mitral valve disease between January 1, 2010, and December 31, 2018, at the Department of Cardiac Surgery, Zhangzhou Affiliated Hospital of Fujian Medical University. Patient data were extracted from our prospectively maintained electronic clinical database, surgical records, and echocardiographic imaging archives.

Inclusion criteria were: (1) preoperative diagnosis of mild functional TR, adjudicated in accordance with contemporary American Society of Echocardiography guidelines [14], which emphasize a comprehensive integrative approach over reliance on any single parameter. The final severity grade was based on the overall assessment of all available echocardiographic data. This integrative approach required the confluence of the following criteria: a vena contracta width (VCW) <3 mm and/or an effective regurgitant orifice area (EROA) <0.2 cm^2^, in combination with a systolic jet area occupying <10% of the right atrial area and no evidence of systolic flow reversal in the hepatic veins; (2) preoperative echocardiographic evidence of tricuspid annular dilation, defined as a diastolic TAD >40 mm measured in the apical 4-chamber view at end-diastole [15]; (3) availability of complete preoperative clinical and echocardiographic data; and (4) availability of at least one comprehensive postoperative transthoracic echocardiogram (TTE) performed ≥1 year after surgery for follow-up assessment.

Exclusion criteria included: (1) moderate or severe TR preoperatively; (2) organic TR etiology (e.g., rheumatic leaflet thickening, Ebstein’s anomaly, carcinoid heart disease, endocarditis, or pacemaker lead-related); (3) concomitant cardiac surgical procedures other than mitral valve surgery (e.g., coronary artery bypass grafting, aortic valve surgery, or atrial fibrillation ablation alone were excluded to ensure a homogeneous cohort focused on the impact of isolated MV procedure); (4) prior history of tricuspid valve surgery or percutaneous intervention; (5) preoperative implantation of a permanent pacemaker or implantable cardioverter-defibrillator; and (6) incomplete key perioperative or follow-up data.

**Fig. 1.**
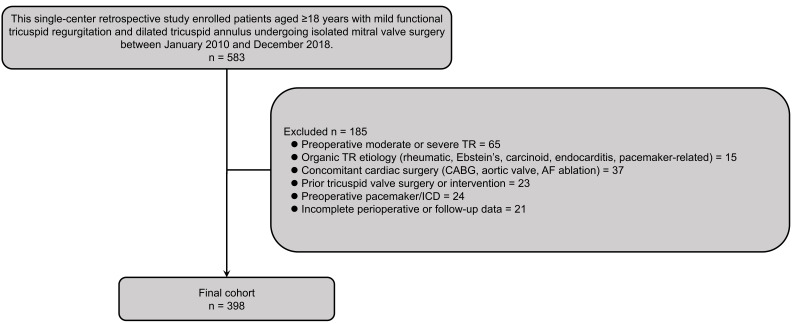
**Study population flowchart**. This flowchart delineates the patient selection process for this retrospective cohort study. From an initial pool of 583 patients who underwent isolated mitral valve surgery between January 2010 and December 2018, 185 patients were excluded based on predefined criteria: preoperative moderate or severe TR (n = 65), organic TR etiology (n = 15), concomitant cardiac surgery (n = 37), prior tricuspid valve intervention (n = 23), preoperative pacemaker/ICD (n = 24), and incomplete perioperative or follow-up data (n = 21). The final analytical cohort comprised 398 patients with mild functional TR and dilated annulus (>40 mm) who met all inclusion criteria. TR, tricuspid regurgitation; CABG, coronary artery bypass grafting; AF, atrial fibrillation; ICD, implantable cardioverter-defibrillator.

A total of 583 patients were initially identified. After applying the exclusion criteria (n = 185), 398 patients formed the final study cohort. A detailed flowchart of patient selection is presented in Fig. 1.

### 2.2 Data Collection and Definitions

Baseline demographic characteristics, clinical history, preoperative comorbidities, and surgical data were systematically collected from electronic medical records by two independent investigators blinded to the postoperative outcomes. Discrepancies were resolved through consensus or by adjudication from a third senior investigator.

Preoperative echocardiographic parameters were obtained from the last comprehensive TTE performed within 3 months before surgery. All preoperative echocardiograms were re-analyzed offline by two experienced cardiologists in a dedicated core laboratory using standard two-dimensional (2D) transthoracic echocardiography. To assess reproducibility, inter- and intra-observer variability were evaluated in a random subset of 50 patients. Inter-observer intraclass correlation coefficients (ICCs) were: TAD index, 0.942 (95% confidence interval [CI]: 0.892–0.972); vena contracta (VC), 0.882 (95% CI: 0.798–0.932); PASP, 0.913 (95% CI: 0.847–0.953); left atrial volume index (LAVI), 0.926 (95% CI: 0.876–0.958). Intra-observer ICCs were: TAD index, 0.961 (95% CI: 0.923–0.981); VC, 0.901 (95% CI: 0.832–0.943); PASP, 0.934 (95% CI: 0.873–0.965); LAVI, 0.948 (95% CI: 0.908–0.972), indicating excellent agreement. Measurements were averaged over three cardiac cycles for patients in sinus rhythm and over five cycles for those in atrial fibrillation. The following parameters were recorded: (1) TR severity: Graded as none, mild, moderate, or severe based on an integrative approach incorporating VCW, EROA (using the proximal isovelocity surface area method when feasible), jet area, hepatic vein flow pattern, and qualitative assessment. (2) TAD: Measured in the apical 4-chamber view at end-diastole from the insertion of the anterior leaflet to the insertion of the posterior leaflet. The TAD index was calculated by dividing TAD by body surface area (BSA). (3) RV size and function: RV basal diameter (RVbase) was measured in the apical 4-chamber view. RV systolic function was assessed by tricuspid annular plane systolic excursion (TAPSE). (4) PASP: Estimated from the peak TR jet velocity using the modified Bernoulli equation and adding an estimated right atrial pressure based on inferior vena cava diameter and collapsibility. (5) Left Ventricular Ejection Fraction (LVEF): Calculated using the biplane Simpson’s method. (6) LAVI: Left atrial volume was measured using the biplane area-length method and indexed to BSA.

Surgical data included the type of MV procedure (repair vs. replacement), surgical approach (conventional sternotomy vs. minimally invasive), and cardiopulmonary bypass (CPB) time. Concomitant procedures specifically related to the tricuspid valve were not performed as per the study’s inclusion criteria of isolated MV surgery.

Postoperative and follow-up data included serial echocardiographic examinations, clinical outcomes, hospital readmissions for heart failure (HF), need for reoperation on the tricuspid valve, and mortality. Follow-up data were obtained through outpatient clinic visits, telephone interviews, and review of national death records, ensuring a comprehensive capture of endpoints.

### 2.3 Study Endpoints

The primary endpoint was postoperative progression of TR, defined as an increase in TR severity to moderate or greater (≥moderate TR) on any follow-up echocardiogram after the index surgery. This was adjudicated by the core laboratory echocardiographers based on the predefined criteria mentioned above [14].

Secondary endpoints included: (1) HF rehospitalization was strictly defined as an unplanned hospitalization >24 hours where new or worsening HF was the primary diagnosis, corroborated by documented symptoms and signs of HF, and requiring administration of intravenous diuretics, vasoactive agents, or mechanical circulatory support, consistent with the 2022 AHA/ACC/HFSA Guideline [16]; (2) reoperation for significant TR, defined as any surgical or percutaneous intervention specifically targeted at the tricuspid valve; (3) all-cause mortality; and (4) new-onset or persistent atrial fibrillation (AF) during follow-up, documented by electrocardiogram or Holter monitoring.

### 2.4 Statistical Analysis

Continuous variables are presented as mean ± standard deviation (SD) if normally distributed or as median with interquartile range (IQR) if non-normally distributed. Normality was assessed using the Shapiro-Wilk test. Categorical variables are expressed as frequencies and percentages (%). Baseline characteristics were compared between the progression group (patients who reached the primary endpoint) and the non-progression group using the Student’s *t*-test or Mann-Whitney U test for continuous variables, and the Chi-square test or Fisher’s exact test for categorical variables, as appropriate.

Univariable logistic regression models were used to assess the association between potential predictors and TR progression. Variables with a *p*-value < 0.1 in the univariable analysis and clinically relevant factors were entered into a multivariable logistic regression model using a stepwise selection method. Candidate predictors were pre-specified based on pathophysiology and literature, including AF type, TAD index, PASP, LAVI, and other clinical/echocardiographic variables. Stepwise selection (entry *p* < 0.1, retention *p* < 0.05) was applied, with final model predictors consuming 6 degrees of freedom (AF type: 3 df; continuous predictors: 1 df each). Results are reported as odds ratios (OR) with 95% CI. Variables with a *p*-value < 0.1 in univariable analysis were considered for inclusion in the multivariable model. It is noteworthy that some variables significant in univariable analysis may not be retained in the final multivariable model due to collinearity, confounding, or because their predictive information is captured by other stronger, pathophysiologically relevant predictors. The final logistic regression model for the probability (P) of TR progression is defined by the equation: Logit(P) = β_0_ + (β_1_ × AF_paroxysmal) + (β_2_ × AF_persistent) + (β_3_ × AF_permanent) + (β_4_ × TADi) + (β_5_ × PASP) + (β_6_ × LAVI), where Logit(P) = ln[P / (1 – P)]. Atrial fibrillation (AF) type, a categorical predictor, was incorporated into the model using dummy coding with “No AF” (Sinus Rhythm) as the reference category. The three derived dummy variables are: AF_paroxysmal (1 if paroxysmal AF, 0 otherwise), AF_persistent (1 if persistent AF, 0 otherwise), and AF_permanent (1 if permanent AF, 0 otherwise). Continuous predictors (TAD index, PASP, LAVI) were scaled per 1 mm/m^2^, 5 mmHg, and 5 mL/m^2^, respectively, and were entered as linear terms after confirming the assumption of linearity in the log-odds using restricted cubic splines with 4 knots (*p* for non-linearity > 0.05 for all). Multicollinearity was evaluated by calculating the variance inflation factor (VIF) for the final model predictors. Model performance was assessed using the area under the curve (AUC) for discrimination, with the 95% CI calculated via the DeLong method. Internal validation was performed using bootstrap resampling with 1000 repetitions to obtain optimism-corrected estimates of model performance and to quantify any overfitting. The following metrics were calculated from the bootstrap procedure: the optimism-corrected AUC with its 95% CI, calibration-in-the-large (intercept), calibration slope, Brier score, and the observed-to-expected (O/E) ratio.

All statistical tests were two-tailed, and a *p*-value < 0.05 was considered statistically significant. All analyses were performed using R software (version 4.4.0, R Foundation for Statistical Computing, Vienna, Austria) and SPSS Statistics (version 26.0, IBM Corp., Armonk, NY, USA).

## 3. Results

### 3.1 Study Population and Baseline Characteristics

A total of 583 patients who underwent isolated mitral valve surgery between January 2010 and December 2018 were initially screened. After applying stringent exclusion criteria, 398 patients with mild functional TR and tricuspid annular dilation (TAD >40 mm) were included in the final cohort (Fig. 1). The cohort was stratified into two groups based on TR progression during follow-up: the non-progression group (n = 279, 70.1%) and the progression group (n = 119, 29.9%), defined as an increase to moderate or severe TR.

Baseline Preoperative Clinical, Echocardiographic and Operative Characteristics are summarized in Table 1. The two groups were well-balanced in terms of age, BSA, etiology of mitral valve disease, New York Heart Association (NYHA) functional class, and comorbidities such as hypertension, diabetes, and chronic obstructive pulmonary disease (COPD) (all *p* > 0.05). However, significant differences were observed in gender distribution (*p* = 0.046), preoperative AF types (*p* < 0.001), TAD (47.75 ± 2.34 mm vs. 42.44 ± 1.45 mm, *p* < 0.001), TAD index (25.56 ± 2.75 mm/m^2^ vs. 22.65 ± 2.24 mm/m^2^, *p* < 0.001), VC (4.82 ± 0.41 mm vs. 3.75 ± 0.42 mm, *p* < 0.001), PASP (58.15 ± 6.91 mmHg vs. 39.97 ± 5.65 mmHg, *p* < 0.001), right ventricular basal (RVbase) (44.06 ± 2.22 mm vs. 37.90 ± 1.72 mm, *p* < 0.001), TAPSE (15.97 ± 1.18 mm vs. 19.11 ± 1.15 mm, *p* < 0.001), and LAVI (68.81 ± 8.37 mL/m^2^ vs. 52.31 ± 7.51 mL/m^2^, *p* < 0.001). No significant differences were noted in mitral valve procedure type, surgical approach, or cardiopulmonary bypass time (all *p* > 0.05).

**Table 1.** **Baseline preoperative clinical, echocardiographic and operative characteristics**.

Subgroup		Non-progression group (n = 279)	Progression group (n = 119)	*p* value
Age (years), Mean ± SD		59.56 ± 8.95	58.94 ± 8.37	0.523
Gender, n (%)				0.046
	Male	150 (53.76)	51 (42.86)	
	Female	129 (46.24)	68 (57.14)	
BSA (m^2^), Mean ± SD		1.89 ± 0.17	1.88 ± 0.18	0.782
Etiology, n (%)				0.728
	Degenerative	95 (34.05)	43 (36.13)	
	Other	93 (33.33)	42 (35.29)	
	Rheumatic	91 (32.62)	34 (28.57)	
NYHA Preop, n (%)				0.509
	I	92 (32.97)	45 (37.82)	
	II	92 (32.97)	40 (33.61)	
	III	95 (34.05)	34 (28.57)	
Hypertension, n (%)				0.353
	Yes	150 (53.76)	70 (58.82)	
Diabetes, n (%)				0.869
	Yes	82 (29.39)	34 (28.57)	
COPD, n (%)				0.429
	Yes	58 (20.79)	29 (24.37)	
Preop AF, n (%)				<0.001
	None	193 (69.18)	35 (29.41)	
	Paroxysmal	26 (9.32)	29 (24.37)	
	Permanent	36 (12.90)	25 (21.01)	
	Persistent	24 (8.60)	30 (25.21)	
Preop TAD (mm), Mean ± SD		42.44 ± 1.45	47.75 ± 2.34	<0.001
Preop TAD index (mm/m^2^), Mean ± SD		22.65 ± 2.24	25.56 ± 2.75	<0.001
Preop VC (mm), Mean ± SD		3.75 ± 0.42	4.82 ± 0.41	<0.001
Preop EROA (cm^2^), Mean ± SD		0.13 ± 0.04	0.12 ± 0.06	0.865
Preop RVol (mL), Mean ± SD		22.71 ± 4.45	22.61 ± 4.39	0.834
Preop PASP (mmHg), Mean ± SD		39.97 ± 5.65	58.15 ± 6.91	<0.001
RVbase (mm), Mean ± SD		37.90 ± 1.72	44.06 ± 2.22	<0.001
Preop TAPSE (mm), Mean ± SD		19.11 ± 1.15	15.97 ± 1.18	<0.001
Preop LVEF (%), Mean ± SD		60.29 ± 5.83	59.74 ± 5.48	0.375
Preop LAVI (mL/m^2^), Mean ± SD		52.31 ± 7.51	68.81 ± 8.37	<0.001
MV procedure, n (%)				0.174
	Replacement	134 (48.03)	66 (55.46)	
	Repair	145 (51.97)	53 (44.54)	
Surgical approach, n (%)				0.293
	Open	159 (56.99)	61 (51.26)	
	Minimally invasive	120 (43.01)	58 (48.74)	
CPB time (min), Mean ± SD		138.16 ± 23.53	141.24 ± 23.08	0.231

SD, standard deviation; BSA, body surface area; NYHA Preop, New York Heart Association Class Preoperative; COPD, chronic obstructive pulmonary disease; TAD, tricuspid annular diameter; VC, vena contracta; EROA, effective regurgitant orifice area; RVol, regurgitant volume; PASP, pulmonary artery systolic pressure; RVbase, Right Ventricular Base; TAPSE, tricuspid annular plane systolic excursion; LVEF, left ventricular ejection fraction; LAVI, left atrial volume index; MV, mitral valve; CPB, cardiopulmonary bypass. For binary variables, only the data for the ‘Yes’ category are displayed. The ‘No’ category serves as the reference. Data are presented as Mean ± Standard Deviation, or Number (%). There were no missing data for the variables presented in this table.

### 3.2 Postoperative Follow-up Echocardiographic Data and Clinical Outcomes

During a median follow-up of 5.2 years (IQR: 3.1–7.4 years), patients in the progression group exhibited significantly larger TAD (52.14 ± 2.89 mm vs. 41.00 ± 1.68 mm, *p* < 0.001), wider VC (6.86 ± 0.67 mm vs. 3.22 ± 0.53 mm, *p* < 0.001), and higher PASP (68.49 ± 7.95 mmHg vs. 36.04 ± 6.02 mmHg, *p* < 0.001) compared to the non-progression group (Table 2). Additionally, the progression group had higher rates of heart failure rehospitalization (26.05% vs. 0%, *p* < 0.001), tricuspid valve reoperation (8.40% vs. 0%, *p* < 0.001), all-cause mortality (2.52% vs. 0%, *p* = 0.026), and new or persistent AF (63.03% vs. 35.48%, *p* < 0.001). The absence of HF rehospitalization and all-cause mortality in the non-progression group aligns with their favorable hemodynamic profile, successful correction of the primary mitral pathology, and the stringent, guideline-based endpoint definitions employed.

**Table 2.** **Postoperative follow-up echocardiographic data and clinical outcomes**.

Subgroup		Non-progression group (n = 279)	Progression group (n = 119)	*p* value
Follow-up TAD (mm), Mean ± SD		41.00 ± 1.68	52.14 ± 2.89	<0.001
Follow-up VC (mm), Mean ± SD		3.22 ± 0.53	6.86 ± 0.67	<0.001
Follow-up PASP (mmHg), Mean ± SD		36.04 ± 6.02	68.49 ± 7.95	<0.001
TRGrade, n (%)				<0.001
	Mild	254 (91.04)	0 (0.00)	
	Moderate	0 (0.00)	51 (42.86)	
	None	25 (8.96)	0 (0.00)	
	Severe	0 (0.00)	68 (57.14)	
HF rehospitalization, n (%)				<0.001
	Yes	0 (0.00)	31 (26.05)	
Reop, n (%)				<0.001
	Yes	0 (0.00)	10 (8.40)	
Death, n (%)				0.026
	Yes	0 (0.00)	3 (2.52)	
AF, n (%)				<0.001
	None	180 (64.52)	44 (36.97)	
	Paroxysmal	28 (10.04)	25 (21.01)	
	Permanent	44 (15.77)	25 (21.01)	
	Persistent	27 (9.67)	25 (21.01)	

TRGrade, Tricuspid Regurgitation Grade; Reop, Reoperation. For binary variables, only the data for the ‘Yes’ category are displayed. The ‘No’ category serves as the reference. Data are presented as Mean ± Standard Deviation, or Number (%). There were no missing data for the variables presented in this table.

### 3.3 Predictors of TR Progression: Univariate and Multivariate Analysis

Univariate logistic regression identified several variables associated with TR progression, including female gender, preoperative AF (all types), TAD, TAD index, VC, PASP, TAPSE, and LAVI (all *p* < 0.05) (Table 3). Variables with *p* < 0.1 in univariate analysis were incorporated into a multivariable logistic regression model. Four independent predictors were identified: preoperative AF type (paroxysmal, OR: 2.764, 95% CI: 1.682–4.532; persistent, OR: 3.422, 95% CI: 2.081–5.625; permanent, OR: 2.345, 95% CI: 1.404–3.917; all *p* < 0.001), TAD index (per 1 mm/m^2^ increase: OR: 2.531, 95% CI: 1.767–3.649, *p* < 0.001), PASP (per 5 mmHg increase: OR: 3.246, 95% CI: 2.191–4.800, *p* < 0.001), and LAVI (per 5 mL/m^2^ increase: OR: 1.876, 95% CI: 1.287–2.733, *p* = 0.001). The final model included four predictors (6 df) with 119 events, yielding an events per variable (EPV) of 19.8, well above recommended thresholds. Although female sex was associated with TR progression in univariate analysis (OR: 1.550, *p* = 0.047), it was not retained as an independent predictor in the multivariable model, suggesting its effect may be mediated through other integrated pathophysiological variables. Multicollinearity assessment for the final model predictors yielded VIF values all below 2.2 (AF Type: 1.24; TAD Index: 1.92; PASP: 2.15; LAVI: 2.01), indicating no significant collinearity.

**Table 3.** **Univariate and multivariate predictors of TR progression (logistic regression analysis)**.

Subgroup		Univariate				Multivariate			
		OR	95% CI	*p* value	β (SE)	OR	95% CI	*p* value	β (SE)
Age (per 5-year increase), Mean ± SD		0.992	0.968–1.017	0.522	–0.008 (0.010)				
Gender, n (%)									
	Male	REF		REF					
	Female	1.550	1.006–2.390	0.047	0.438 (0.220)				
BSA (per 0.1 m^2^ increase), Mean ± SD		0.839	0.243–2.893	0.781	–0.175 (0.625)				
Etiology, n (%)									
	Degenerative	REF		REF					
	Other	0.998	0.598–1.666	0.993	–0.002 (0.010)				
	Rheumatic	0.825	0.484–1.408	0.481	–0.192 (0.012)				
NYHA Preop, n (%)									
	I	REF		REF					
	II	0.889	0.531–1.487	0.654	–0.118 (0.015)				
	III	0.732	0.431–1.243	0.248	–0.312 (0.018)				
Hypertension, n (%)									
	No	REF		REF					
	Yes	1.228	0.795–1.898	0.353	–0.206 (0.221)				
Diabetes, n (%)									
	No	REF		REF					
	Yes	0.961	0.598–1.543	0.869	0.040 (0.230)				
COPD, n (%)									
	No	REF		REF					
	Yes	1.228	0.738–2.041	0.429	–0.206 (0.261)				
Preop AF, n (%)									
	None	REF		REF		REF		REF	
	Paroxysmal	6.151	3.242–11.667	<0.001	1.817 (0.205)	2.764	1.682–4.532	<0.001	1.017 (0.253)
	Persistent	6.893	3.612–13.155	<0.001	1.930 (0.215)	3.422	2.081–5.625	<0.001	1.230 (0.254)
	Permanent	3.829	2.051–7.151	<0.001	1.342 (0.210)	2.345	1.404–3.917	<0.001	0.852 (0.262)
Preop TAD (per 5 mm increase)		13.065	5.826–29.301	<0.001	2.569 (0.412)				
Preop TAD index (per 1 mm/m^2^ increase)		1.589	1.428–1.769	<0.001	0.463 (0.085)	2.531	1.767–3.649	<0.001	0.929 (0.185)
Preop VC (per 2 mm increase)		3.000	2.109–4.271	<0.001	1.099 (0.180)				
Preop EROA (per 0.1 cm^2^ increase)		0.658	0.005–8.115	0.865	–0.418 (0.035)				
Preop Rvol (per 10 mL increase)		0.995	0.948–1.044	0.834	–0.005 (0.025)				
Preop PASP (per 5 mmHg increase)		1.965	1.601–2.412	<0.001	0.675 (0.110)	3.246	2.191–4.800	<0.001	1.177 (0.200)
RVbase (per 5 mm increase)		2.961	0.890–9.859	0.791	0.916 (0.614)				
Preop TAPSE (per 2 mm increase)		0.042	0.017–0.101	<0.001	–3.170 (0.941)				
Preop LVEF (per 5% increase)		0.983	0.947–1.021	0.374	–0.017 (0.015)				
Preop LAVI (per 5 mL/m^2^ increase)		1.343	1.257–1.436	<0.001	0.295 (0.070)	1.876	1.287–2.733	0.001	0.629 (0.192)
MV procedure, n (%)									
	Replacement	REF		REF					
	Repair	0.742	0.482–1.142	0.175	–0.298 (0.225)				
Surgical approach, n (%)									
	Open	REF		REF					
	Minimally invasive	1.260	0.819–1.938	0.293	0.231 (0.220)				
CPB time (per 10 min increase)		1.006	0.996–1.015	0.230	0.006 (0.005)				

Data are presented as Mean ± Standard Deviation, or Number (%). OR, odds ratio; CI, confidence interval; SE, standard errors; REF, reference.

### 3.4 Predictive Model Development and Clinical Application

**Fig. 2.**
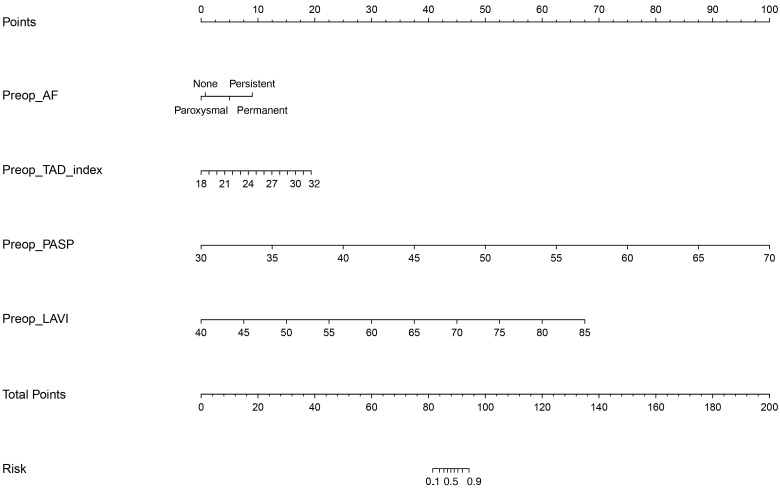
**Nomogram for predicting the risk of TR progression after isolated mitral valve surgery**. The nomogram integrates four independent predictors identified by multivariable logistic regression: preoperative AF type, TAD index, PASP, and LAVI. Each variable value corresponds to a point score on the top axis. The sum of these points corresponds to a total points value, which is then projected downward to the ‘Risk’ axis to estimate the predicted probability of TR progression. Worked Example: To manually calculate the risk for a patient with Persistent AF (dummy variable AF_persistent = 1, AF_paroxysmal = 0, AF_permanent = 0), a TADi of 26 mm/m^2^, a PASP of 60 mmHg, and an LAVI of 72 mL/m^2^, use the model equation: Logit(P) = –7.832 + (1.230 × 1) + (0.929 × 26) + (1.177 × [60 / 5]) + (0.629 × [72 / 5]) = 40.734. The predicted probability P = e^^(40.734)^ / (1 + e^^(40.734)^) ≈ 1.00 (>99.9%), indicating a very high risk consistent with the nomogram projection.

Based on the results of the multivariable analysis, a predictive nomogram was constructed to quantify the individualized risk of TR progression after isolated mitral valve surgery (Fig. 2). This visual tool integrates the four validated predictors: preoperative AF type, TAD index, PASP, and LAVI. For each patient, points are assigned for each variable value on the top axis. The sum of these points yields a total point score, which is then projected downward to the bottom axis to directly read the predicted probability of TR progression. This nomogram facilitates rapid bedside or pre-operative clinic estimation of risk, aiding in patient counseling and informing discussions about the potential need for concomitant tricuspid valve intervention at the time of initial mitral surgery.

The corresponding logistic regression equation with the final coefficients is: Logit(P) = –7.832 + (1.017 × AF_paroxysmal) + (1.230 × AF_persistent) + (0.852 × AF_permanent) + (0.929 × TADi) + (1.177 × [PASP / 5]) + (0.629 × [LAVI / 5]), where TADi is in mm/m^2^, PASP in mmHg, and LAVI in mL/m^2^. The intercept (β_0_) is –7.832. This equation allows for the direct calculation of the predicted probability: P = e^^L⁢o⁢g⁢i⁢t⁢(P)^ / (1 + e^^L⁢o⁢g⁢i⁢t⁢(P)^).

### 3.5 Model Validation and Performance Metrics

**Fig. 3.**
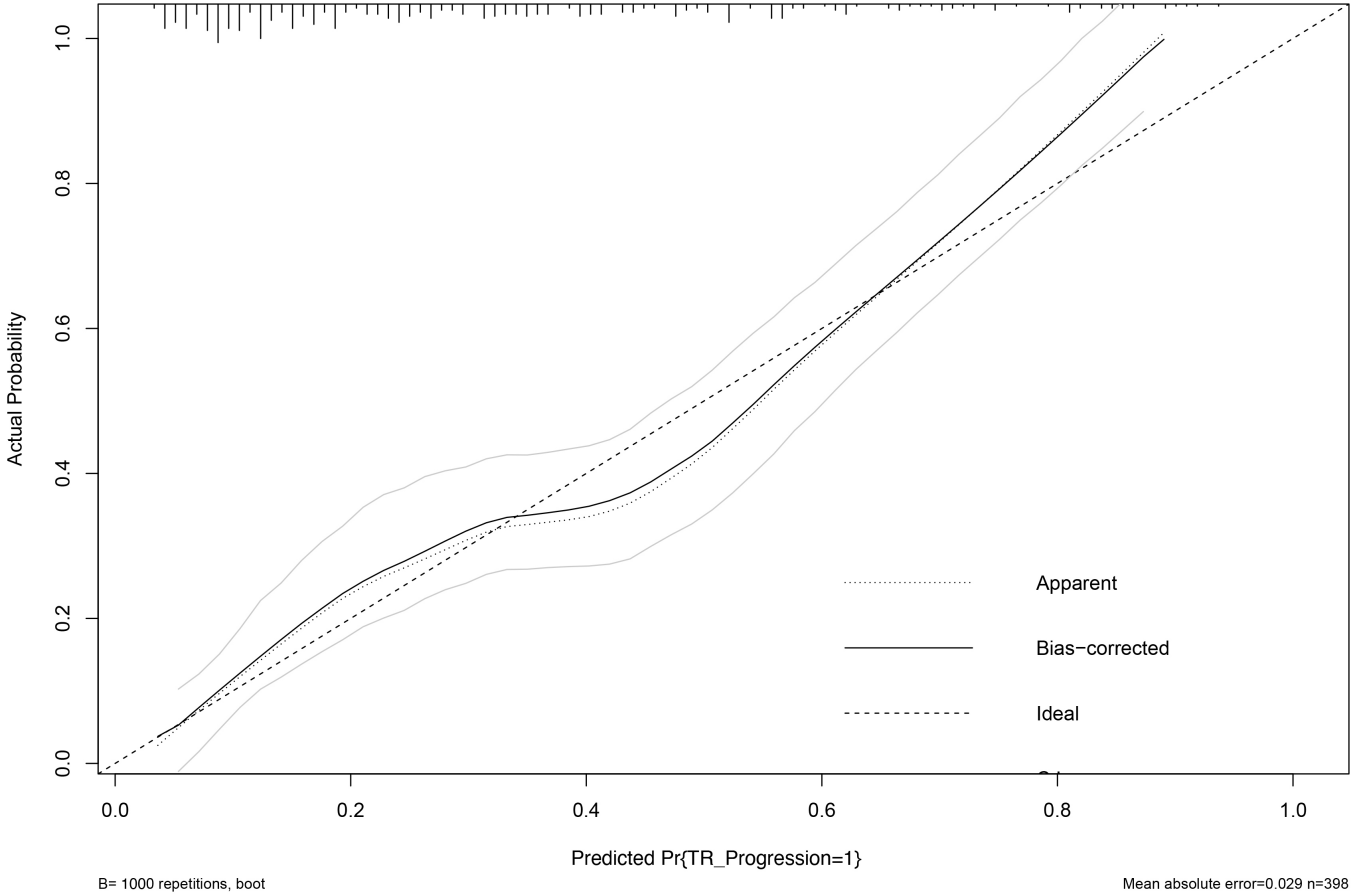
**Optimism-corrected calibration curve of the nomogram for predicting TR progression**. The calibration plot depicts the agreement between predicted probabilities and observed event frequencies after internal validation with 1000 bootstrap resamples. The solid line represents the bias-corrected (optimism-corrected) calibration curve, which closely aligns with the ideal line of perfect agreement, indicating excellent calibration. The corresponding optimism-corrected calibration metrics are: calibration slope = 0.94, calibration-in-the-large (intercept) = –0.03, and Brier score = 0.152.

The calibration of the prediction model was rigorously assessed through internal validation with 1000 bootstrap resamples. This procedure yielded optimism-corrected performance metrics, demonstrating excellent calibration: calibration-in-the-large (intercept) = –0.03, calibration slope = 0.94, Brier score = 0.152, and an observed-to-expected (O/E) ratio of 0.98. The corresponding optimism-corrected calibration curve (Fig. 3) closely aligns with the line of perfect agreement, visually confirming minimal overfitting.

**Fig. 4.**
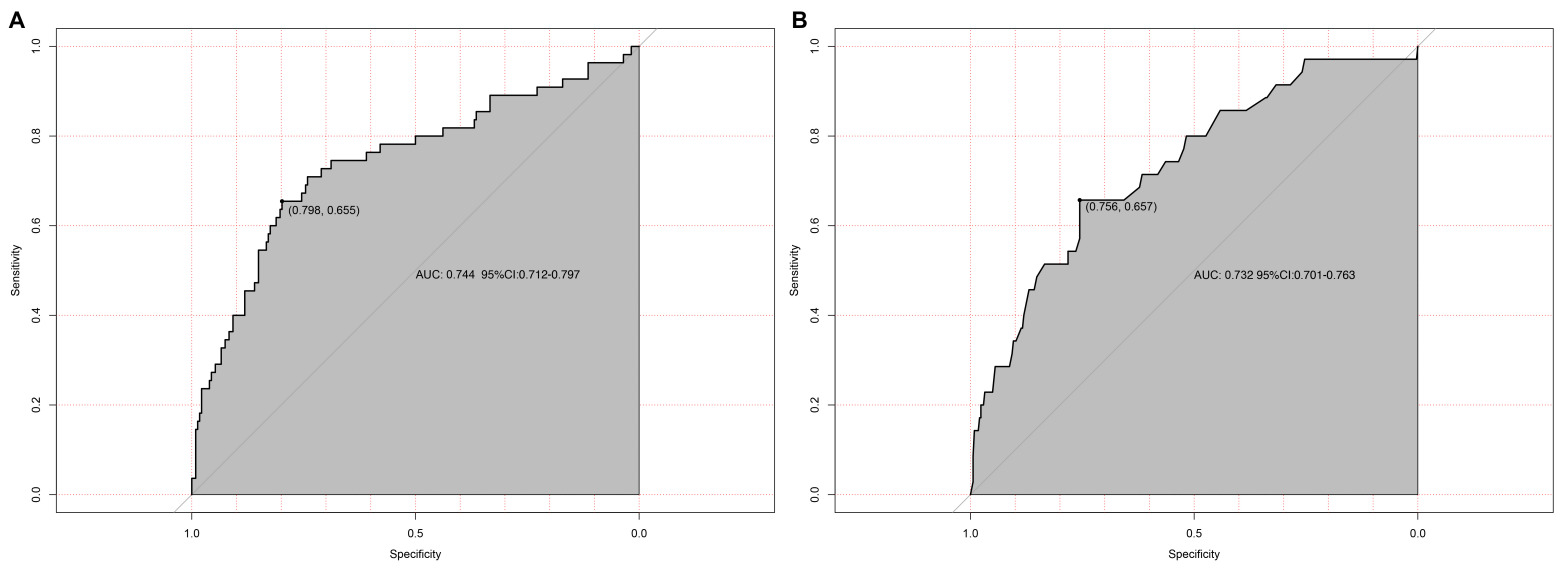
**Discriminatory performance of the TR progression prediction model**. (A) Apparent receiver operating characteristic (ROC) curve in the derivation cohort (n = 398). The area under the curve (AUC) is 0.744 (95% CI: 0.712–0.797; DeLong method). (B) Optimism-corrected ROC curve after internal validation using 1000 bootstrap resamples, yielding a validated AUC of 0.732 (95% CI: 0.701–0.763). The shaded bands represent the 95% CI.

The model’s discriminatory ability was evaluated using a receiver operating characteristic (ROC) curve (Fig. 4). The apparent AUC in the derivation cohort was 0.744 (95% CI: 0.712–0.797, DeLong method). Internal validation via 1000 bootstrap resamples yielded an optimism-corrected AUC of 0.732 (95% CI: 0.701–0.763), confirming stable discriminatory performance with minimal overfitting.

### 3.6 Clinical Validation and Decision Threshold Analysis

**Fig. 5.**
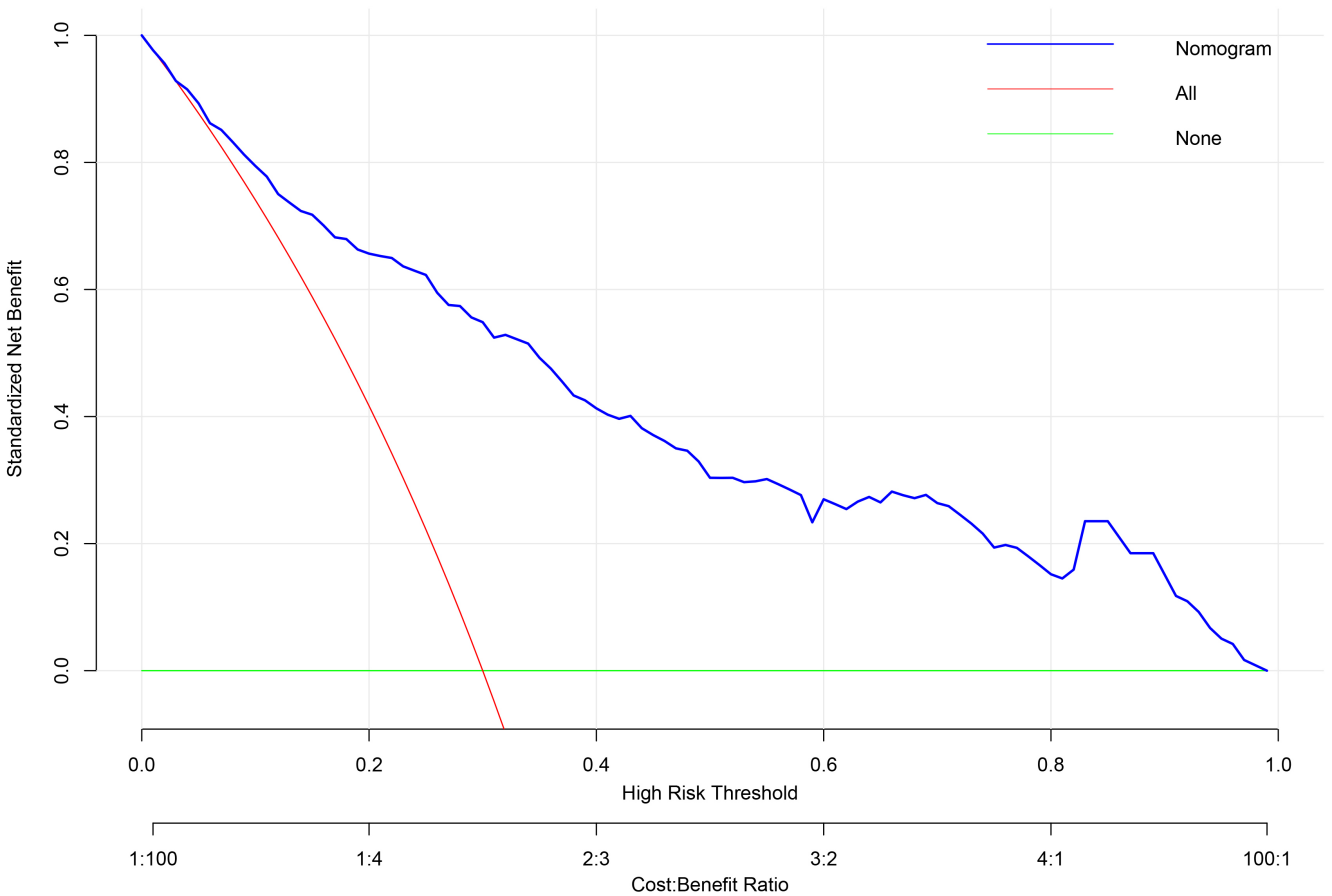
**Decision curve analysis (DCA) for the TR progression prediction model**. DCA evaluates the clinical utility of the nomogram across a range of threshold probabilities (top x-axis). The net benefit (y-axis) of the model (solid blue line) is compared against strategies of treating all patients (solid red line) or none (solid green line). The model shows superior net benefit within the clinically relevant threshold probability range of 0.2–0.7. The Threshold Probability represents the minimum probability of disease progression at which a clinician would opt for intervention. It is conceptually linked to the relative weight of the harm of an unnecessary intervention (cost) versus the benefit of correctly treating a patient who would progress (benefit), where Threshold Probability = Cost / (Cost + Benefit). The corresponding Cost: Benefit Ratio is shown on the bottom x-axis.

DCA was performed to evaluate the clinical utility of the nomogram across a range of threshold probabilities (Fig. 5). DCA compares the net benefit of using the model to guide clinical decisions versus alternative strategies (e.g., intervening in all or no patients). The nomogram provided superior net benefit compared to the “treat-all” or “treat-none” strategies within the threshold probability range of 20% to 70%. The optimal threshold was identified at 45%, where the net benefit peaked.

At this 45% threshold, the model demonstrated a sensitivity of 72.3% (95% CI: 63.5–79.8%), specificity of 76.0% (95% CI: 70.6–80.8%), a positive predictive value of 58.1% (95% CI: 49.8–66.0%), and a negative predictive value of 85.7% (95% CI: 80.8–89.6%). The net benefit was 0.185 (95% CI: 0.150–0.220), significantly exceeding alternative strategies. Calibration was excellent at this cut-point, with an observed event rate of 46.2% in the predicted risk range of 40–50%.

This threshold facilitates a direct translation to clinical action: for patients with a predicted risk ≥45%, concomitant tricuspid intervention is strongly recommended, as the long-term benefit of preventing TR progression outweighs the incremental procedural risk. For patients with a predicted risk <45%, isolated mitral valve surgery without tricuspid repair is advised, coupled with intensified postoperative surveillance to detect potential progression. Thus, the 45% threshold enables a personalized, risk-stratified strategy—targeting proactive intervention to the ~30% of patients at highest risk while sparing the majority unnecessary procedure-related risks.

**Fig. 6.**
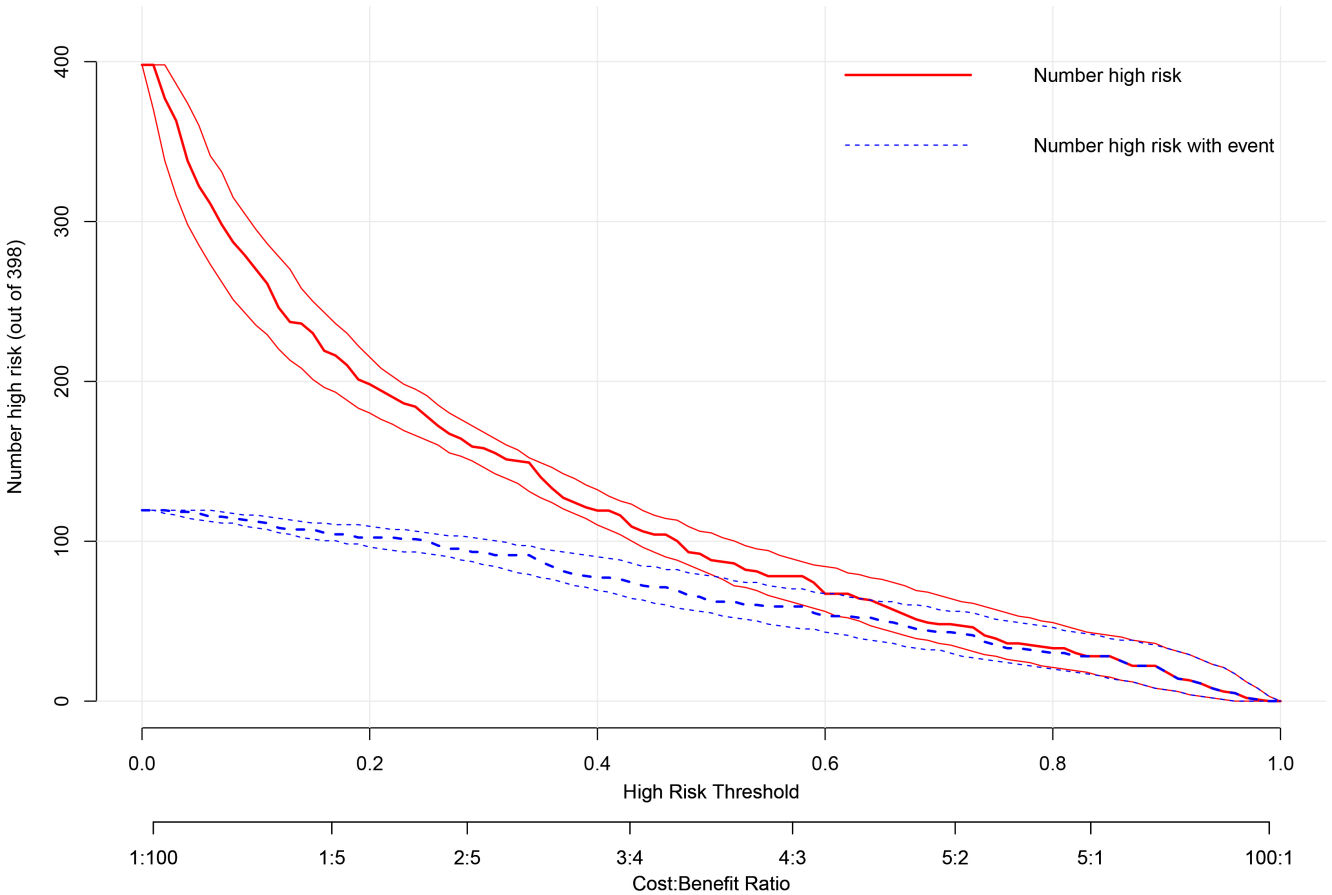
**Clinical impact curve (CIC) of the prediction model**. The CIC displays the number of patients classified as high-risk (red line) and the number of true events (TR progression) among them (blue line) across increasing risk thresholds (x-axis). At the optimal threshold of 0.45, the model identifies the majority of true progressors while maintaining a manageable number of false positives, effectively balancing clinical benefit against intervention costs.

Clinical impact curve (CIC) analysis (Fig. 6) further illustrated the balance between true positives and false positives across risk thresholds. At the 45% threshold, the model identified the majority of true progressors while maintaining a manageable false-positive rate, supporting its potential for optimizing resource allocation and individualized patient management.

## 4. Discussion

### 4.1 Key Findings and Clinical Implications

This study successfully developed and internally validated a novel, pathophysiology-integrated nomogram for individualized prediction of TR progression in patients with mild functional TR and annular dilation undergoing isolated mitral valve surgery. The model incorporates four readily available preoperative variables—AF type, TAD index, PASP, and LAVI—which collectively capture the key hemodynamic, anatomic, and remodeling substrates of FTR. The model demonstrated good discriminative capacity (AUC: 0.744, 95% CI: 0.712–0.797) and, crucially, exceptional calibration and clinical utility across a spectrum of risk thresholds, as rigorously assessed by decision curve and clinical impact analyses.

Our findings address a pivotal uncertainty in contemporary valvular heart disease management. While current guidelines recommend concomitant tricuspid surgery for severe FTR and suggest it for milder degrees with annular dilation (TAD ≥40 mm or >21 mm/m^2^) [7], this anatomic criterion alone lacks precision for predicting which patients with mild FTR will progress post-operatively. This can result in both overtreatment, exposing patients to the inherent risks of a more complex procedure without clear benefit, and undertreatment, missing the opportunity to prevent debilitating right heart failure and the high risk of reoperation. The presented nomogram facilitates a paradigm shift from a one-size-fits-all anatomic strategy to a personalized, risk-adjusted approach. By identifying the ~30% of patients at high risk (e.g., predicted risk >45%), heart teams can selectively recommend prophylactic tricuspid surgery, potentially averting adverse long-term outcomes. Conversely, confidently avoiding tricuspid surgery in the majority of low-risk patients spares them the incremental risks of heart block, pacemaker implantation, and prolonged cardiopulmonary bypass, thereby optimizing the collective risk-benefit ratio of mitral valve surgery.

### 4.2 Comparison With Prior Research and Methodological Advances

Our model advances the field beyond previous prediction efforts, which often focused on isolated parameters or lacked robust validation [17, 18]. It synergizes multiple dimensions of FTR pathophysiology: the arrhythmic substrate (AF type), the anatomic substrate (TADi), the hemodynamic driver (PASP), and the marker of chronic left atrial pressure overload (LAVI). This integrated approach aligns with the modern understanding of FTR as a progressive biventricular disorder intricately linked to left-sided heart disease and atrial myopathy [10, 19], rather than a simple consequence of annular dilation.

The biological plausibility of each predictor is well-established. Preoperative AF, particularly non-paroxysmal forms, contributes to bi-atrial enlargement, annular dilation, and RV dysfunction, creating a persistent substrate for TR despite correction of the mitral lesion [20, 21, 22]. TADi provides a body size-adjusted measure of the fundamental anatomic derangement. PASP is a direct quantifier of RV afterload, a primary driver of RV remodeling and tricuspid leaflet tethering that may not fully normalize after MV surgery, especially if long-standing [3, 23, 24, 25]. LAVI is a robust, integrative marker of the chronicity and severity of left atrial hypertension, the initial insult in the pathophysiological cascade leading to pulmonary hypertension and subsequent FTR [26, 27, 28]. Our multivariate model captures the interplay of these factors, offering a more holistic risk assessment.

Methodologically, this study adheres to Transparent Reporting of a multivariable prediction model for Individual Prognosis Or Diagnosis guidelines for transparent reporting and employs advanced techniques for rigorous internal validation [29]. The use of bootstrapping corrects for over-optimism, and the application of DCA is a critical strength, moving beyond traditional discriminative metrics to quantify the clinical net benefit of using the model for decision-making across a range of patient and physician risk tolerances [30]. This analysis confirms that the nomogram provides superior clinical utility compared to current strategies for a broad range of threshold probabilities.

### 4.3 Clinical Translation and Real-World Applicability

The proposed nomogram is designed for straightforward integration into the heart team’s preoperative workflow, utilizing data (AF status, echocardiographic measures) that are routinely and universally available. The DCA indicates that employing this model to guide the decision for concomitant tricuspid surgery provides a net benefit compared to alternative strategies (treat-all or treat-none) for threshold probabilities between approximately 20% and 70%, with an optimum around 45%. This threshold represents a rational balance where the benefits of preventing TR progression likely outweigh the risks of adding a prophylactic procedure.

Implementing this risk-based strategy could yield substantial clinical and economic impacts. For high-risk patients, proactive tricuspid surgery could avert the detrimental sequelae of progressive TR, improving functional capacity, quality of life, and survival, while obviating the high risks and costs associated with reoperation [31, 32]. For healthcare systems, preventing disease progression reduces long-term heart failure management costs. Furthermore, by safely avoiding tricuspid surgery in a majority of patients, the model could reduce immediate procedural morbidity and resource utilization, enhancing overall healthcare value.

### 4.4 Limitations and Future Directions

Several limitations must be acknowledged. First, the retrospective, single-center design, while employing rigorous statistical correction, is susceptible to unmeasured confounding and limits generalizability. Specifically, our cohort reflects the patient population and surgical practice of a single tertiary center, which may introduce referral bias and affect the direct applicability of our absolute risk estimates to other settings. Furthermore, the requirement for a follow-up echocardiogram at least one year post-operatively, while ensuring data quality for the primary endpoint, may introduce survivorship bias by excluding patients who died or were lost to follow-up early. This could under-represent the highest-risk patients, potentially affecting the model’s predicted probabilities for those with imminent early mortality, although the identified pathophysiological associations are likely robust. Second, the model was derived and validated in a cohort exclusively undergoing isolated MV surgery for FTR; patients undergoing concomitant procedures, such as surgical atrial fibrillation ablation (which was an exclusion criterion to isolate the effect of the MV procedure), were not represented. Thus, its performance in patients with other etiologies of FTR or those requiring combined procedures remains unknown. Third, echocardiographic measurements, though performed by a core lab with excellent reproducibility, possess inherent variability.

These limitations delineate a clear path for future research. The immediate priority is the external validation of our nomogram. Subsequently, a prospective randomized trial comparing a nomogram-guided strategy versus current guideline-based management would provide the highest level of evidence for its clinical efficacy. Further investigation is warranted to explore whether modulating these risk factors (e.g., aggressive rhythm control for AF, pulmonary vasodilators) can alter the natural history of TR progression. Integration of novel imaging biomarkers, such as cardiac magnetic resonance imaging, could further enhance predictive precision and provide deeper mechanistic insights [33].

In conclusion, we have developed and internally validated a pathophysiology-based predictive model that quantifies individual risk for TR progression after isolated MV surgery. This tool provides a pragmatic and evidence-based framework to guide the contentious decision regarding concomitant tricuspid intervention, aiming to optimize long-term outcomes for patients with functional tricuspid regurgitation.

## 5. Conclusions

In conclusion, we have developed and internally validated a pathophysiology-integrated nomogram that accurately predicts the risk of TR progression in patients with mild functional TR and annular dilation following isolated MV surgery. The model, incorporating four readily available preoperative variables—AF type, TAD index, PASP, and LAVI—demonstrated robust discriminative ability, excellent calibration, and meaningful clinical utility across a range of risk thresholds. This tool facilitates a paradigm shift from a one-size-fits-all anatomic approach to a personalized, risk-stratified strategy, enabling heart teams to selectively recommend concomitant tricuspid intervention for high-risk patients while avoiding unnecessary procedures in those at low risk. By optimizing surgical decision-making, our nomogram holds significant promise for improving long-term clinical outcomes in this challenging patient population.

## Availability of Data and Materials

The data supporting this study’s findings are available within the article. Raw datasets containing protected health information are restricted to preserve patient confidentiality. De-identified data may be provided to qualified researchers upon reasonable request to the corresponding author, subject to ethics approval and data sharing agreements.
